# Trauma system developments reduce mortality in hospitalized trauma patients in Al-Ain City, United Arab Emirates, despite increased severity of injury

**DOI:** 10.1186/s13017-020-00327-y

**Published:** 2020-08-18

**Authors:** David O. Alao, Arif Alper Cevik, Hani O. Eid, Zia Jummani, Fikri M. Abu-Zidan

**Affiliations:** 1 Department of Internal Medicine, College of Medicine and Health Sciences, UAE University, Al-Ain, United Arab Emirates; 2 Emergency Department, Al-Ain Hospital, Al-Ain, United Arab Emirates; 3 Department of Emergency Medicine, Mediclinic Middle East, Airport Road Hospital, Abu Dhabi, United Arab Emirates; 4 Department of Surgery, College of Medicine and Health Sciences, UAE University, Al-Ain, United Arab Emirates

**Keywords:** Injury, Trauma, Prevention, Mechanism, Intervention, Incidence

## Abstract

**Background:**

Trauma is a leading cause of death in the United Arab Emirates (UAE). There have been major developments in the trauma system in Al-Ain City during the last two decades. We aimed to study the effects of these developments on the trauma pattern, severity, and clinical outcome of hospitalized trauma patients in Al-Ain City, United Arab Emirates.

**Methods:**

This is a retrospective analysis of two separate sets of prospectively collected trauma registry data of Al-Ain Hospital. Data were collected over two periods: from March 2003 to March 2006 and from January 2014 to December 2017. Demography, injury mechanism, injury location, and clinical outcomes of 2573 trauma patients in the first period were compared with 3519 patients in the second period.

**Results:**

Trauma incidence decreased by 38.2% in Al-Ain City over the last 10 years. Trauma to females, UAE nationals, and the geriatric population significantly increased over time (*p* < 0.0001, Fisher’s exact test for each). Falls on the same level significantly increased over time, while road traffic collisions and falls from height significantly decreased over time (*p* < 0.0001, Fisher’s exact test for each). Mortality significantly decreased over time (2.3% compared with 1%, *p* < 0.0001, Fisher’s exact test).

**Conclusions:**

Developments in the trauma system of our city have reduced mortality in hospitalized trauma patients by 56% despite an increased severity of injury. Furthermore, the injury incidence in our city decreased by 38.2% over the last decade. This was mainly in road traffic collisions and work-related injuries. Nevertheless, falls on the same level in the geriatric population continue to be a significant problem that needs to be addressed.

## Background

Trauma is a leading cause of mortality worldwide, causing 5.8 million deaths annually [1]. In 2017, it caused 17.2% of all deaths in the United Arab Emirates (UAE). Road traffic collisions and falls cause about 44% of these deaths [2].

Al-Ain City is located in the Emirate of Abu Dhabi, UAE. It has a population of 766,009, consisting of 30% UAE nationals and 70% non-national inhabitants. The majority of non-nationals are laborers [3]. Al-Ain Hospital is a general secondary teaching hospital with a capacity of 450 beds. It is a designated trauma center which receives the majority of trauma patients in our city.

There have been major developments in the trauma system in Al-Ain City over the last two decades. In 2001, a Trauma Group was established with the mission of promoting research and education to international standards so as to improve patient care. Al-Ain Hospital trauma registry was established in 2003 and was operated for a few years until it was suspended in 2007 due to lack of public funding [4]. Studies based on records from this registry were used to inform injury prevention strategies and promote the establishment of a trauma system [5, 6].

As a result of these developments, numerous injury-prevention interventions were introduced. These included installation of a large number of road speed cameras, imposition of high penalties for speeding violations, educational and awareness programs on speeding and vehicle safety devices, and the enforcement of work safety regulations [7–9]. Meanwhile, the educational activities of the Trauma Group included establishing and running the Advanced Trauma Life Support program (ATLS) [10] and Focussed Assessment Sonography for Trauma Courses [11, 12]. Furthermore, major improvements in the prehospital transport system and trauma management were led by the Department of Health of Abu-Dhabi. The number of health care providers and EMS-trained staff increased [13], principles of damage control surgery were adopted [12, 14], and 24-h interventional radiology became available.

Given the broad range of these developments, it is beneficial to evaluate their impact on trauma pattern and clinical outcome in Al-Ain Hospital using the data available from our two trauma registries. These data were available for the historical period 2003–2006 and for the re-established trauma registry starting from 2014. We aimed to study the effects of the improvements in the trauma system on the trauma pattern, severity, and clinical outcome of hospitalized trauma patients in Al-Ain City, United Arab Emirates.

## Patients and methods

### Ethical considerations

Ethical approval for this study was obtained from the Human Research Ethics Committee of Al-Ain Hospital, Al-Ain, United Arab Emirates (AAHEC-03-20-008). Written informed consent to use patients’ data for this research study was taken from the patients or their caregivers.

### Data collection

This is a retrospective analysis of two separate sets of prospectively collected trauma registry data. All trauma patients who were hospitalized in Al-Ain Hospital for more than 24 h or who died on arrival at the hospital were included. Data from two periods were analyzed: March 2003 to March 2006, and January 2014 to December 2017.

### Studied variables

Studied variables included age, gender, nationality, mechanism and location of injury, method of transportation, physiological and anatomical severity markers (systolic blood pressure, heart rate, respiratory rate), Injury Severity Score (ISS), New Injury Severity Score (NISS), Glasgow Coma Scale (GCS), ICU admission, length of hospital stay, and clinical outcome. We categorized nationality into two groups: UAE and non-UAE, because we have previously shown that risks of injury for these two groups are different in our city [15].

### Calculations

Al-Ain City had an estimated population of 460,000 during the first study period [14] and has a current population of 766,009 inhabitants [6]. Al-Ain Hospital is a designated trauma center which treats about 80% of the hospitalized trauma patients of the city. Accordingly, the standardized incidence of hospitalized trauma patients per 100,000 population in Al-Ain City was calculated as follows: (1.25 × annual admissions)/(population/100000).

### Statistical analysis

Data were presented as mean (SD) for continuous data, median (range) for ordinal data, or number (%) for categorical data. Pearson’s Chi square or Fisher’s exact test was used to compare categorical data of two independent groups. For large X × Y tables, overall significance of the table was tested. If the overall analysis was significant, then pairwise comparisons were done to explain the findings. Mann-Whitney *U* test was used to compare continuous or ordinal data for independent groups. Statistical analyses were performed using the Statistical Package for the Social Sciences (IBM-SPSS version 26, Chicago, Il). A *p* value of less than 0.05 was accepted as significant.

## Results

During the first period, an average of 858 patients was admitted to Al-Ain Hospital for trauma per year, whereas an average of 880 patients was admitted per year for trauma during the second period. The estimated annual incidence of hospitalized trauma patients in Al-Ain City was 233 per 100,000 population for the first period, compared with 144 per 100,000 population for the second period, a reduction of 38.2% in trauma incidence over approximately 10 years.

**Fig. 1:**
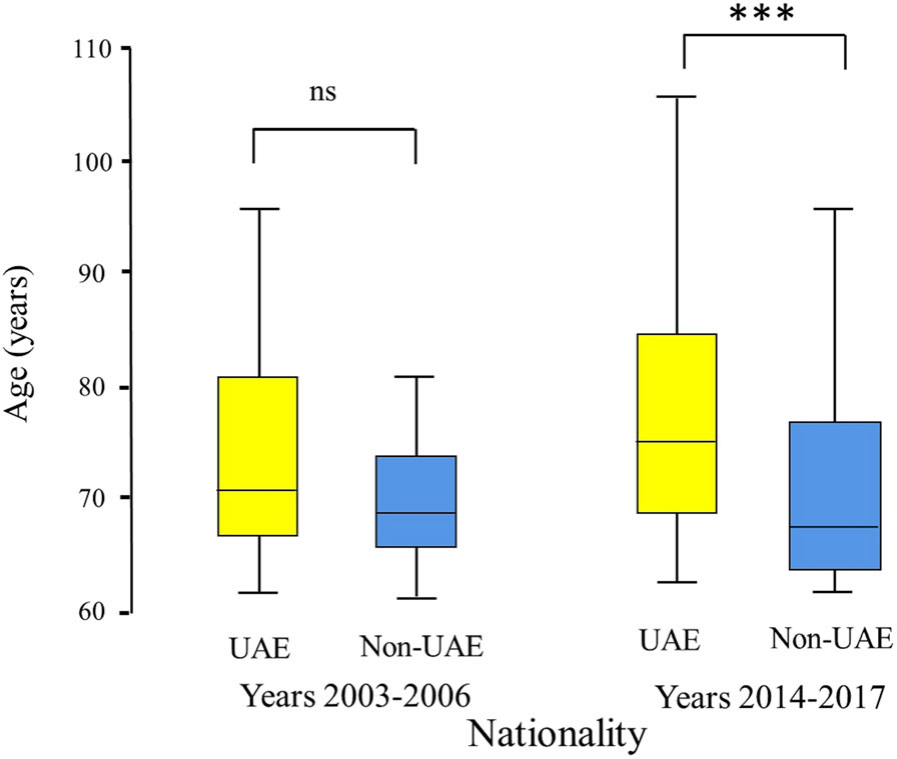
Box-and-whisker plot of age (years) of hospitalized geriatric trauma patients (older than 60 years) by nationality during the period 2003–2006 (*n* = 79) and 10 years later (*n* = 263) during the period 2014–2017, Al-Ain Hospital, Al-Ain, United Arab Emirates. The box represents the 25th to the 75th percentile IQR. The horizontal line within each box represents the median. ns, non-significant, ***p < 0.0001, Mann-Whitney *U* test

Table 1 compares the demography of patients and severity of injury for the two periods. The percentage of both females and of UAE nationals who suffered injuries has significantly increased over time (females 17.9% compared with 21.2%, *p* = 0.002; and UAE Nationals 13.4% compared with 17.6%, *p* < 0.0001 respectively). The geriatric group has significantly increased over time from 3.1 to 7.5% of the population (*p* < 0.0001). During the second period, UAE nationals were significantly older in the geriatric group (above 60 years old) compared with non-UAE nationals (median (range) 74 (61–105) years compared with median (range) 68 (61–100) years, *p* < 0.0001), but this difference was not significant in the first period (median (range) age of 70 (61–95) years compared with median (range) of 68 (61–100) years, *p* = 0.06) **(**Fig. 1**)**.

**Table 1:** Demography and severity of injury of hospitalized patients during the period 2003–2006 (*n* = 2573) and 10 years later (*n* = 3519) during the period 2014–2017, Al-Ain Hospital, Al-Ain, United Arab Emirates

Variable	Years 2003–2006	Years 2014–2017	*p* value
Age	31.4 (15.1)	32.9 (17.9)	0.12
Age group			< 0.0001
< 18 years	419 (16.3%)	610 (17.3%)	
18–60 years	2059 (80.5%)	2646 (75.2%)	
> 60 years	79 (3.1%)	263 (7.5%)	
Gender			< 0.0001
Male	2228 (86.6%)	2901(82.4%)	
Female	345 (13.4%)	618 (17.6%)	
UAE nationals	461 (17.9%)	745 (21.2%)	0.002
By ambulance	863 (33.5%)	1330 (37.8%)	< 0.001
SBP	133.1 (21.7)	135 (31.7)	< 0.0001
Heart rate	90.3 (18.7)	89.4 (20)	0.002
Respiratory rate	21 (4.2)	19.3 (3.9)	< 0.0001
GCS^a^	15 (3–5), 14.34 (2.48)	15 (3–5), 14.74 (1.44)	< 0.0001
ISS^a^	4 (1–43), 5.61 (6)	4 (1–75), 6.48 (6.21)	< 0.0001
NISS	4 (1–75)	6 (1–75)	< 0.0001
ICU admission	202 (7.9%)	559 (6.3%)	0.02
Hospital stay	9.2 (12.6)	5.9 (7.5)	< 0.0001
Death	58 (2.3%)	35 (1%)	<0.0001

*SBP* systolic blood pressure, *GCS* Glasgow coma scale, *ISS* injury severity score, *NISS* new injury severity score

*p* = Pearson Chi square or Fisher’s exact test as appropriate for categorical data and Mann Whitney *U* test for ordinal or continuous data

^a^Data are presented as mean (SD), median (range), or number (%) as appropriate. Ordinal data are occasionally presented in addition as mean (SD) if the median is the same

**Fig. 2:**
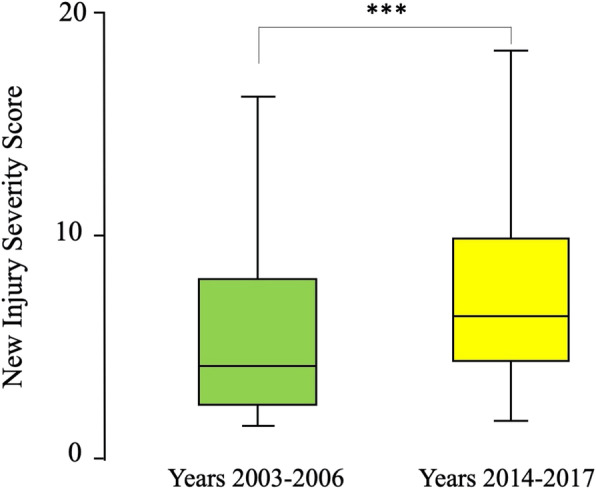
Box-and-whisker plot of New Injury Severity Score (NISS) for hospitalized trauma patients during the period 2003–2006 (*n* = 2573) and 10 years later (*n* = 3519) during the period 2014–2017, Al-Ain Hospital, Al-Ain, United Arab Emirates. The box represents the 25th to the 75th percentile IQR. The horizontal line within each box represents the median. ***p < 0.0001, Mann-Whitney *U* test

The percentage of patients who were brought to the Emergency Department by ambulance increased from 33.5 to 37.8% (*p* < 0.0001). During the first period, patients who arrived at the Emergency Department had significantly lower systolic blood pressure (mean (SD) 133.1 (21.7) mmHg compared with 135 (31.7) mmHg, *p* < 0.0001), had more tachycardia (mean (SD) 90.3 (18.7) mmHg compared with 89.4 (20) beat per minute, *p* = 0.002), and lower GCS (mean (SD) 14.34 (2.48) compared with 14.74 (1.44), *p* < 0.0001). Nevertheless, the ISS and NISS were significantly higher in the second period (median (range) NISS 4 (1–75) compared with 6 (1–75), *p* < 0.0001) **(**Fig. 2**)**. The ICU admission, length of hospital stay, and percentage of deaths significantly decreased over time (7.9% compared with 6.3%, *p* = 0.02; mean (SD) 9.2 (12.6) days compared with 5.9 (7.5) days, *p* < 0.0001; and 2.3% compared with 1%, *p* < 0.0001 respectively).

Table 2 shows the mechanism of injury in the two periods. Motor vehicle collisions, falls from a height, injuries from falling heavy objects, and bicycle injuries significantly decreased over time (36% compared with 26.2%, *p* < 0.0001; 18.4% compared with 12%, *p* < 0.0001; 6.1% compared with 4.5%, *p* = 0.008; and 3% compared with 1.7%, *p* < 0.001, respectively). In contrast, falling down and machinery injuries significantly increased over time (15.9% compared with 26.6%, *p* < 0.0001; and 4% compared with 5.5%, *p* = 0.005 respectively). There was no change in the proportion of motorcycle injuries and burns. Fall injuries were significantly more frequent among geriatric patients (237/342 (69.2%) compared with others 1106/5734 (19.3%), *p* < 0.0001, Pearson’s Chi Square).

**Table 2:** Comparison of mechanism of injury of hospitalized patients during the period 2003–2006 (*n* = 2573) and 10 years later (*n* = 3519) during the period 2014–2017, Al-Ain Hospital, Al-Ain, United Arab Emirates

Mechanism	Years 2003–2006		Years 2014–2017		
	Number	%	Number	%	*p* value
MVC	925	36	921	26.2	< 0.0001
Fall from height	473	18.4	422	12	< 0.0001
Fall down	409	15.9	936	26.6	< 0.0001
Heavy object	156	6.1	160	4.5	0.008
Burn	145	5.6	173	4.9	0.21
Machinery	102	4	195	5.5	0.005
Bicycle	76	3	59	1.7	< 0.001
Motorcycle	68	2.6	94	2.7	0.94
Other	219	8.5	559	15.9	< 0.0001
Total	2573	100	3519	100	

*p* = Pearson Chi square or Fisher’s exact test as appropriate

Table 3 shows the location where injuries occurred. There was a significant drop in trauma occurring on streets and highways, off roads, work places, and farms (40% compared with 28.7%, *p* < 0.0001; 5.4% compared with 2.8%, *p* < 0.0001; 29.5% compared with 21.6%, *p* < 0.0001; and 3.2% compared with 1%, *p* < 0.0001 respectively). In contrast, injuries in the home and in public places significantly increased (19.6% compared with 39%, *p* < 0.0001; and 1% compared with 5.8%, *p* < 0.0001 respectively).

**Table 3:** Comparison of location of occurrence of injury of hospitalized patients during the period 2003–2006 (*n* = 2573) and 10 years later (*n* = 3519) during the period 2014–2017, Al-Ain Hospital, Al-Ain, United Arab Emirates

Location	Years 2003–2006		Years 2014–2017		
	Number	%	Number	%	*P* value
Street/highway	1028	40.0	989	28.7	< 0.0001
Work-place	757	29.5	746	21.6	< 0.0001
Home	503	19.6	1346	39.0	< 0.0001
Off-road	140	5.4	98	2.8	< 0.0001
Farm	82	3.2	34	1.0	< 0.0001
Public area	26	1.0	201	5.8	< 0.0001
Other	34	1.3	37	1.1	0.37
Total	2570	100.0	3451	100.0	

*p* = Pearson Chi square or Fisher’s exact test as appropriate

Numbers do not add to the total number of each period because of missing data

## Discussion

Our study has shown that developments in the trauma system of Al-Ain city have reduced mortality in hospitalized trauma patients by 56% and reduced injury incidence by 38% over the last decade. Road traffic collisions, falls from height, and injuries from falling objects significantly decreased, which could all be attributed largely to injury prevention interventions. Nevertheless, falls on the same level in the geriatric population remain a significant problem.

Trauma system establishment reduces mortality by 25% in severely injured patients [16]. The 56% decrease in mortality in our study occurred despite the increased injury severity of our admitted patients, as indicated by the ISS and ICU admissions. These encouraging data are supported by the World Health Organization reports. The death rate resulting from road traffic collisions in the UAE dropped from 37 per 100,000 population in 2004 to 11.6 per 100,000 population in 2018 (68.6% decrease over 14 years) [17, 18]. The number of patients transported by ambulance to the Emergency Department has significantly increased. The improved vital signs of injured patients on arrival at the Emergency Department in the second period reflect an overall improvement in EMS pre-hospital care. EMS training and accreditation have expanded and improved in Abu Dhabi Emirate [19].

Trauma is a major cause of death in developing countries globally [20]. We think that the experience of developing a trauma system in Al-Ain City is unique and important. This required investment in the infrastructure, modernization of the health care system, training and education, and understanding of the epidemiology of injury, combined with enforcement of legislation and change in behavior [8, 9, 21, 22].

The percentage of females and UAE nationals who are injured has significantly increased over time. Participation of females in the workforce has increased in the UAE. Over 43% of women have a bachelor’s degree compared with 23% of men. As a result, unemployment among women has dropped from 13% in 2013 to 9.6% in 2017 [23]. This was associated with increased driving and outdoor activities.

Falls have become the first cause of injury at present compared with road traffic collisions 10 years ago. The proportion of geriatric trauma patients increased from 3.1 to 7.5% over the study period. UAE has the highest aging index among all Gulf countries due to improved health care [24]. The increased risk of falls in the geriatric population is related to multiple factors including gait imbalance, weak joints, and use of medications [25]. Falls caused almost 70% of geriatric injuries in the current study which is similar to findings from other studies [26, 27]. The location of injury has significantly changed over time. There was a decrease in injuries occurring on the roads compared with an increase of injuries occurring in the home and in public places. We need to develop a strategy to address injury prevention in these two locations.

## Limitations

We have to acknowledge that there are certain limitations in our study. *First*, there was a gap in our registry between 2007 and 2014 because of lack of funding. *Second*, we studied trauma patients only in Al-Ain City which may not reflect the whole of the UAE. Nevertheless, our city is a small city which gives us an excellent opportunity to evaluate the effects of interventions on trauma epidemiology and management. *Third*, these hospitalized patients represent the tip of the iceberg of injuries as our study does not address those trauma patients who do not present at the hospital. Al-Ain Hospital trauma registry was the only one available in the UAE until 2010, so we could only use these data for comparison in our study. *Fourth*, we cannot define exactly the impact of each component of the trauma system individually. *Finally*, there are no detailed data on the underlying cause of injury or the associated circumstances.

## Conclusions

Developments in the trauma system of our city have reduced mortality in hospitalized trauma patients by 56% despite the increased severity of injury. Furthermore, the incidence of injury in our city was reduced by 38.2% over the last decade. This was mainly a result of a decrease in injuries caused by road traffic collisions and of work-related injuries. Nevertheless, falls on the same level in the geriatric population continue to be a significant problem that needs to be addressed.

## Data Availability

There is no additional data available to share with the readers. Data can be shared with the Editor of the Journal if requested.

## Abbreviations

ATLS: Advanced Trauma Life Support program
GCS: Glasgow Coma Score
ICU: Intensive care unit
ISS: Injury Severity Score
NISS: New Injury Severity Score
SBP: Systolic blood pressure
SD: Standard deviation
UAE: United Arab Emirates

## References

[1] World Health Organization. Injuries and violence: the facts. WHO. 2012. Available on: https://www.who.int/violence_injury_prevention/publications/factsheets/all/en/. Accessed 27 Apr 2020.

[2] Abu Dhabi Health Statistics 2017. Available on: https://www.doh.gov.ae/-/media/Feature/Resources/AbuDhabiHealthStatistics.ashx. Accessed 27 Apr 2020.

[3] Statistics Centre, Abu Dhabi. Available on: https://www.scad.gov.ae. Accessed 27 Apr 2020.

[4] Shaban S, Ashour M, Bashir M, El-Ashaal Y, Branicki F, Abu-Zidan FM (2009). The long term effects of early analysis of a trauma registry. World J Emerg Surg..

[5] Matt Kwong and Suryatapa Bhattacharya. Research highlights danger to labourer The National, October 02, 2009. Available on https://www.thenational.ae/uae/health/research-highlights-danger-to-labourers-1.534744 . Accessed 18 June 2020.

[6] Olivia Olarte. Trauma system in the pipeline in Abu Dhabi. Khaleej Times, October 25, 2011. http://www.khaleejtimes.com/DisplayArticle08.asp?xfile=/data/theuae/2011/October/theuae_October631.xml&section=theuae (Accessed on 18^th^ June 2020).

[7] Grivna M, Aw TC, El-Sadig M, Loney T, Sharif AA, Thomsen J (2012). The legal framework and initiatives for promoting safety in the United Arab Emirates. Int J Inj Control Saf Promot.

[8] United Arab Emirates Government Portal. Available on: https://www.u.ae/en/information-and-services/justice-safety-and-the-law/road-safety. Accessed 05 May 2020.

[9] United Arab Emirates Government Portal. Health and safety at work place. Available on: https://www.u.ae/en/information-and-services/jobs/health-and-safety-at-workplace. Accessed 5 May 2020.

[10] Abu-Zidan FM, Mohammad A, Jamal A, Chetty D, Gautam SC, van Dyke M (2014). Factors affecting success rate of advanced trauma life support (ATLS) courses. World J Surg.

[11] Abu-Zidan FM, Dittrich K, Czechowski JJ, Kazzam EE (2005). Establishment of a course for focused assessment sonography for trauma. Saudi Med J.

[12] Abu-Zidan FM (2016). On table POCUS assessment for the IVC following abdominal packing: how I do it. World J Emerg Surg..

[13] Department of Health Abu Dhabi Health Statistics 2017. Available from: https://www.doh.gov.ae/-/media/Feature/Resources/AbuDhabiHealthStatistics.ashx . Accessed 27 Apr 2020.

[14] Bashir MM, Abu-Zidan FM (2003). Damage control surgery for abdominal trauma. Eur J Surg Suppl.

[15] Grivna M, Eid HO, Abu-Zidan FM (2014). Epidemiology, morbidity and mortality from fall-related injuries in the United Arab Emirates. Scand J Trauma Resusc Emerg Med.

[16] Moore L, Champion H, Tardif PA, Kuimi BL, O’Reilly G, Leppaniemi A (2018). Impact of trauma system structure on injury outcomes: a systematic review and meta-analysis. World J Surg.

[17] World Health Organization. World report on road traffic injury prevention. 2004. Available from: https://www.who.int/publications/i/item/world-report-on-road-traffic-injury-prevention. Accessed 27 Apr 2020.

[18] World Health Organization. Global status report on road safety 2018. [Available from: https://www.who.int/publications-detail/global-status-report-on-road-safety-2018 . Accessed 27 Apr 2020.

[19] Fares S, Irfan FB, Corder RF, Al Marzouqi MA, Al Zaabi AH, Idrees MM (2014). Emergency medicine in the United Arab Emirates. Int J Emerg Med.

[20] Yasin YJ, Grivna M, Abu-Zidan FM (2020). Reduction of pedestrian death rates: a missed global target. World J Emerg Surg.

[21] Barss P, Addley K, Grivna M, Stanculescu C, Abu-Zidan F (2009). Occupational injury in the United Arab Emirates: epidemiology and prevention. Occup Med (Lond).

[22] El-Sadig M, Sarfraz Alam M, Carter AO, Fares K, Al-Taneuiji HO, Romilly P (2004). Evaluation of effectiveness of safety seatbelt legislation in the United Arab Emirates. Accid Anal Prev.

[23] United Arab Emirates Ministry of Economics. Economical reports. 2018. Available on https://www.economy.gov.ae/english/Knowledge-Section/Pages/Economical-Reports.aspx . Accessed 27 Apr 2020.

[24] Khan HTA, Hussein S, Deane J (2017). Nexus between demographic change and elderly care need in the Gulf cooperation council (GCC) countries: some policy implications. Ageing Int.

[25] Gillespie L, Handoll H (2009). Prevention of falls and fall-related injuries in older people. Inj Prev.

[26] Kehoe A, Smith JE, Edwards A, Yates D, Lecky F (2015). The changing face of major trauma in the UK. Emerg Med J.

[27] Dixon JR, Lecky F, Bouamra O, Dixon P, Wilson F, Edwards A (2020). Age and the distribution of major injury across a national trauma system. Age Ageing.

